# Recent Trends in Diabetes-Associated Hospitalizations in the United States

**DOI:** 10.3390/jcm11226636

**Published:** 2022-11-09

**Authors:** Muni Rubens, Venkataraghavan Ramamoorthy, Anshul Saxena, Peter McGranaghan, Elise McCormack-Granja

**Affiliations:** 1 Primary Care, Baptist Health South Florida, Miami, FL 33176, USA; 2 Herbert Wertheim College of Medicine, Florida International University, Miami, FL 33199, USA; 3 Center for Healthcare Advancement and Outcomes, Baptist Health South Florida, Miami, FL 33143, USA; 4 Charité—Universitätsmedizin Berlin, Corporate Member of Freie Universität Berlin and Humboldt Universität zu Berlin, 10117 Berlin, Germany

**Keywords:** diabetes, hospitalization, trend, cost, epidemiology

## Abstract

The purpose of this study was to examine trends in diabetes-related hospitalizations over the period 2010 to 2019 using Nationwide Inpatient Sample (NIS) to facilitate informed policies regarding diabetes-related prevention and management. Between 2010 and 2019, there were 304 million hospitalizations above 18 years of age, of which 78 million were diabetes-associated hospitalizations. The overall population-adjusted diabetes hospitalizations significantly increased from 3079.0 to 3280.8 per 100,000 US population (relative increase, 6.6%, Ptrend < 0.028). Age-stratified analysis showed that hospitalizations significantly increased for 18–29 years (relative increase, 7.8%, Ptrend < 0.001) while age- and gender-stratified analysis showed that diabetes hospitalization significantly increased for 18–29-year males (relative increase, 18.1%, Ptrend < 0.001). Total hospitalization charge increased from 97.5 billion USD in 2010 to 132.0 billion USD in 2019 (relative increase, 35.4%, Ptrend < 0.001). Our study’s findings suggest that diabetes-associated hospitalizations will continue to increase in the future because recent evidence indicates a reappearance of diabetes complications. It is important to screen, prevent, and control diabetes at a younger age based on the trends observed in our study.

## 1. Introduction

Diabetes is a leading cause of morbidity and mortality in the US. According to recent estimates, the prevalence of diabetes is 14.7% among adults aged 18 years or older [1]. The age-standardized prevalence of diabetes increased substantially from 9.8% to 14.8% between 1999–2000 and 2017–2018 [2]. Diabetes is associated with complications such as cardiovascular and cerebrovascular diseases, nephropathy, retinopathy, peripheral neuropathy, nonhealing ulcers, and amputations, and the majority of these complications require intensive treatments [3]. Diabetes is also associated with significant economic burden. For example, in 2017, more than 327 billion USD was spent in managing diabetes, of which 237 billion USD was on direct medical expenditures and 90 billion USD on productivity losses [4].

Diabetes can complicate medical and surgical conditions leading to increased rate and frequency of hospitalizations. Diabetes is also associated with adverse hospital outcomes such as increased rates of complications, length of stay, in-hospital mortality, and higher hospitalization cost [5,6]. A significant majority of these complications require rigorous treatments and readmissions and sustained cost for disability and rehabilitation, even after discharge from hospitalizations. Diabetes-associated hospitalization is also an indirect indicator of the performance of primary care as primary prevention and better glycemic control could potentially prevent the majority of these hospitalizations [7]. A study done by Lee et al. among children and young adults showed that the proportion of younger adults hospitalized with diabetes in the US increased significantly from 1993 to 2004 [8]. Another study found that there were large increases in diabetes hospitalizations among adults aged 30–39 years and young women from 1993 to 2006 [9]. However, recent trends on diabetes hospitalizations are scarce. In this study, we examined the trends in diabetes associated hospitalizations from 2010 to 2019, using the Nationwide Inpatient Sample (NIS) data to facilitate informed policy decisions regarding prevention and management of this condition.

## 2. Materials and Methods

The current study used the NIS database for assessing the trends in diabetes-associated hospitalizations during 2010 to 2019. The Agency for Healthcare Research and Quality (AHRQ) developed the NIS, which constitutes the largest database for all-payer inpatient services in the United States [10]. The NIS contains discharge data from more than 1200 hospitals located in 45 states and has been available yearly since 1988 [10]. The NIS collects stratified samples of approximately 20% of US community hospital discharge data and has been helpful in calculating national estimates. Individual hospitalizations are recorded in the NIS with one primary and as many as 24 secondary diagnoses, after appropriate confidentiality and de-identification procedures to ensure privacy of patients, physicians, and hospitals. Yearly quality assurance procedures are conducted to ensure the internal validity of the database.

The current study was a trend analysis of data from NIS, collected between 2010 and 2019. Both Type 1 and Type 2 diabetes-associated hospitalizations during this period were identified with International Classification of Diseases, 9th Revision, Clinical Modification (ICD-9-CM) and ICD-10-CM codes (Supplementary Table S1). A previous study reported an accuracy of 91% for identifying diabetes from administrative data using ICD codes [11]. All hospitalizations with same admission and discharge dates and total length of stay less than one day were excluded from the analyses because these events would not have truly represented hospital stays.

Statistical analyses were performed using SAS version 9.4 (SAS Inc., Cary, NC, USA), which accounted for the complex survey design and clustering. To improve calculation of national estimates, the NIS was redesigned in 2012. After this period, the NIS collected sample discharge records from all participating hospitals. We used trend weight (TRENDWT) for 2010 and 2011 and regular discharge weight (DISCWT) for 2012 to 2019 to account this change. The checklist for working with the NIS was used to ensure methodological appropriateness for the study [12]. Descriptive statistics were used to compare the demographic characteristics of diabetes-associated hospitalizations between 2010 and 2019. We used the Chi square test for comparing these characteristics. To estimate the significance of trends across the years, survey-weighted linear (PROC REGRESS) and logistic (PROC RLOGIST) regressions were used for continuous and categorical variables, respectively. Relative change% was calculated as an absolute increase or decrease in prevalence between 2010 and 2019. Yearly diabetes-associated hospitalization rates were estimated for 100,000 US population. This was calculated by dividing diabetes-associated hospitalizations by estimated number of US adults aged ≥18 years. Hospitalization rates were also stratified by age groups (18–29 years, 30–39 years, 40–49 years, 50–59 years, 60–69 years, 70–79 years, and ≥80 years) and gender. Inpatient stay costs were calculated by multiplying total hospital charge and cost-to-charge ratio. The costs for each year were adjusted according to 2019 inflation levels released by the US Consumer Price Index [13].

## 3. Results

Between 2010 and 2019, there were 304 million hospitalizations above 18 years of age, of which 78 million were diabetes-associated hospitalizations. During both 2010 and 2019 majority of the hospitalizations for diabetes occurred in the age groups 60–69 and 70–79 years, followed by ≥80 years and 50–59 years. Among these hospitalizations, there was a greater proportion of females (52.2%) during 2010, whereas there was a greater proportion of males during 2019 (51.2%). During both years, the majority of these hospitalizations were among Whites, followed by Blacks, Hispanics, Asians or Pacific Islanders, and Native Americans. Likewise, during both years, the majority of these hospitalizations had Medicare coverage, followed by private insurance, Medicaid, and self-pay. Income distribution during both years showed that the majority of these hospitalizations were among the lowest median household income quartile, followed by the second, third, and highest quartiles. The medians (interquartile range) for length of stay during 2010 and 2019 were 3.1 (1.6–5.8) and 3.2 (1.6–6.0) days, respectively. The prevalence of complications such diabetic retinopathy, diabetic nephropathy, diabetic neuropathy, and lower-extremity amputations decreased, though not significantly, between 2010 and 2019. Comparison of demographic characteristics showed that age groups, gender, and insurance status differed significantly between 2010 and 2019. Table 1 shows the comparison of demographic characteristics of diabetes hospitalizations between 2010 and 2019.

**Figure 1:**
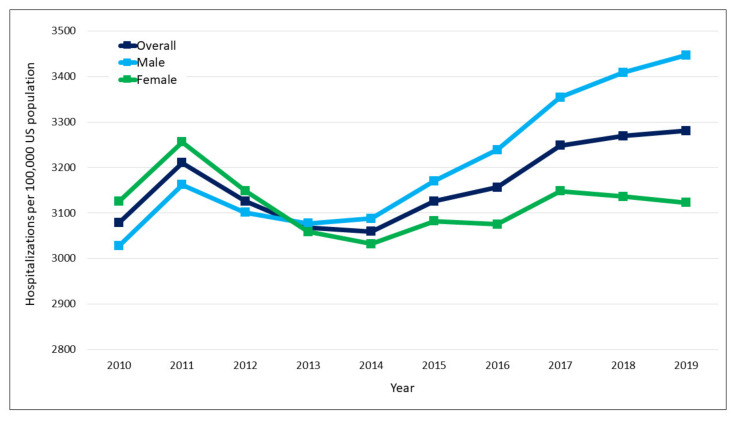
Trends in overall and gender-specific population-adjusted diabetes associated hospitalizations.

**Figure 2:**
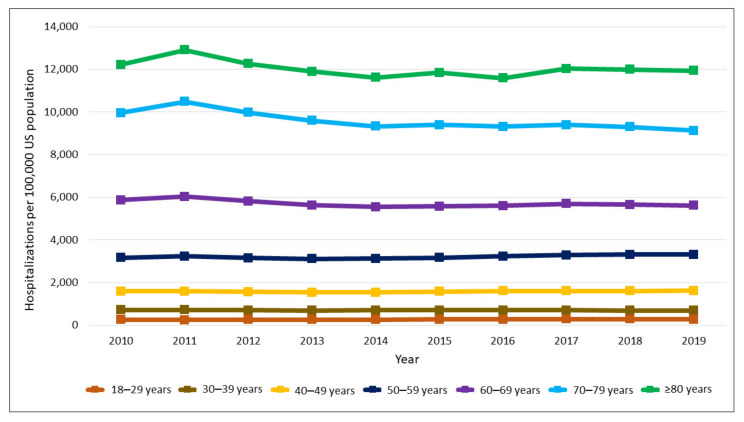
Trends in age-specific population-adjusted diabetes associated hospitalizations.

Trend analysis showed that the overall population-adjusted diabetes hospitalizations significantly increased from 3079.0 to 3280.8 per 100,000 US population (relative increase, 6.6%, Ptrend < 0.028) (Figure 1). Age-stratified analysis showed that hospitalizations significantly increased for 18–29 years (relative increase, 7.8%, Ptrend < 0.001) and 50–59 years (relative increase, 4.7%, Ptrend = 0.019) and significantly decreased for 60–69 years (relative decrease, 4.2%, Ptrend = 0.036), and 70–79 years (relative decrease, 8.4%, Ptrend = 0.002) (Figure 2). However, there were no significant changes for the age groups 30–39 years (relative decrease, 3.6%, Ptrend = 0.082) and 40–49 years (relative increase, 1.8%, Ptrend = 0.098). Gender-stratified analysis showed that trends of diabetes hospitalization increased significantly for males (relative increase, 13.8%, Ptrend < 0.001), but did not show significant difference for females (relative decrease, 0.1%, Ptrend = 0.529) (Figure 1). The top 5 reasons for diabetes hospitalizations in 2010 were diabetes mellitus with complications, coronary artery disease, congestive heart failure, sepsis, and acute kidney failure, and in 2019 were diabetes mellitus with complications, congestive heart failure, coronary artery disease, pneumonia, and cellulitis and abscess of leg. Supplementary Table S2 shows the list of top 10 primary diagnoses among hospitalizations with diabetes in 2010 and 2019.

Age- and gender-stratified analysis showed that diabetes hospitalization increased for 18–29-year males (relative increase, 18.1%, Ptrend < 0.001), 18–29-year females (relative increase, 2.4%, Ptrend = 0.012), 40–49-year males (relative increase, 6.4%, Ptrend = 0.002), and 50–59-year males (relative increase, 10.9%, Ptrend < 0.001). Age- and gender-stratified analysis also showed that diabetes hospitalization decreased for 30–39 years female (relative decrease, 7.3%, Ptrend = 0.001), 60–69 years female (relative decrease, 10.6%, Ptrend < 0.001), 70–79 years male (relative decrease, 3.9%, Ptrend = 0.035), 70–79 years female (relative decrease, 12.9%, Ptrend < 0.001), and ≥80 years female (relative decrease, 6.0%, Ptrend = 0.016). However, there were no significant changes among 30–39 years male (relative increase, 1.5%, Ptrend =0.316), 40–49 years female (relative decrease, 2.8%, Ptrend = 0.237), 50–59 years female (relative decrease, 2.1%, Ptrend = 0.294), 60–69 years male (relative increase, 2.2%, Ptrend = 0.437), and ≥80 years male (relative increase, 1.3%, Ptrend = 0.732). Supplementary Table S3 shows the trends in diabetes-associated hospitalizations stratified by age and gender.

**Figure 3:**
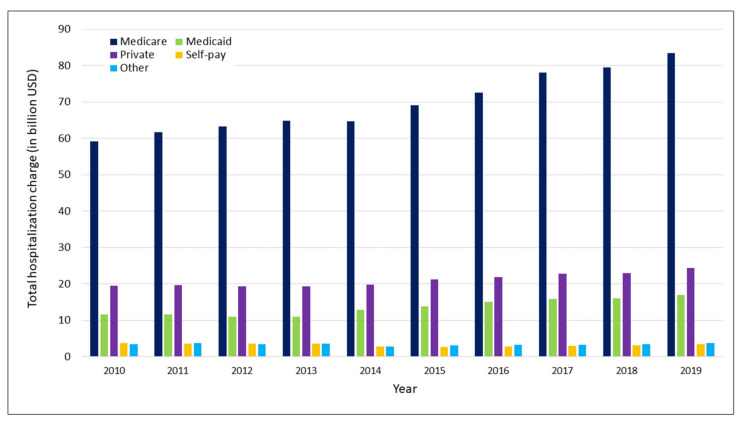
Trends in total diabetes associated hospitalization charges stratified by insurance type.

Total hospitalization charge increased from 97.5 billion USD in 2010 to 132.0 billion USD in 2019 (relative increase, 35.4%, Ptrend < 0.001). In 2019, Medicare (83.5 billion USD) accounted for the largest share of diabetes associated hospital charges, followed by private insurance (24.4 billion USD), and Medicaid (17.0 billion USD) (Figure 3).

## 4. Discussion

In this study, using a national database, we found that diabetes associated hospitalizations significantly increased in the last decade. The most striking finding was the increase observed among the males in the age group 18–29 years. The majority of the increase in diabetes-associated hospitalization rates was driven by alarming levels of increase observed among males in general. We also found that the charges associated with diabetes associated hospitalizations increased significantly during the study period.

The increase in overall diabetes-associated hospitalizations could be attributed to several factors. The most important factor could be the increase in the prevalence of diabetes during the study period. After witnessing substantial improvements in diabetes control during 1999 to 2010, this trend plateaued and deteriorated in the US [14,15]. Changing paradigms in diabetes management guided by evolving evidence based on the debate regarding the requirement for intensive glycemic control could have resulted in this trend. For example, three randomized controlled trials published in 2008 and 2009 showed that intensive glycemic control did not produce any cardiovascular benefit and rather increased the risk for hypoglycemic complications [16,17,18]. These studies prompted the American Diabetes Association (ADA) and the European Association for the Study of Diabetes (EASD) to recommend a more individualized and moderate approach to provide care with more focus on individual patient preferences, requirements, and values [19]. These events were followed by a more conservative approach for glycemic control. For example, a study by Fang and colleagues found that the use of antidiabetic medications increased by 8.6% during 1999 to 2010 and plateaued from 2011 to 2018, and there was a decline in the use of sulfonylureas and thiazolidinediones. This study also found that prescription of single antidiabetic medications increased from 35.6% in 2007–2010 to 40.1% in 2015–2018, whereas prescription of two (29.1% to 27.7%) and three (15.1% to 14.9%) antidiabetic medications decreased during the same period. The early period of our study coincided with major healthcare reforms such as the implementation of the Affordable Care Act (ACA), which expanded insurance coverage to more than 20 million people within the US. Increasing rates of diabetes-associated hospitalizations in our study could have been a direct consequence of these reforms. We observed increasing trends in these hospitalizations after 2013–2014 when ACA started to take effect. ACA had significant effects on diabetic population because health insurance coverages increased, and diabetes medical costs decreased substantially among this population. This also improved the affordability of healthcare services such as ambulatory and inpatient resources, especially among poorer sections. For example, a cross-sectional study using National Health Interview Surveys found that during 2009 to 2016 health insurance coverage increased significantly from 84.7% to 90.1% among diabetic adults aged 18–64 years [20]. Uninsured diabetic patients have worse levels of diabetic control and complications and are less likely to frequent healthcare resources including hospitals [21,22]. The greater availability of coverages within the provisions of ACA would have motivated this section of the population to actively seek treatments and hospitalizations for diabetes and associated complication.

The most alarming finding of our study was the significant increase in diabetes-associated hospitalizations observed among younger adults aged 18–29 years. This is concerning because microvascular and macrovascular changes start early and significantly increase the risk and severity of associated complications as this population ages. This could have long terms effects such as impaired quality of life and increased direct and indirect healthcare expenditures. This increase could be attributed to several factors. The prevalence of diabetes among younger adults increased substantially during the study period [23]. In addition, complications of diabetes such as diabetic ketoacidosis (DKA) and hyperglycemic hyperosmolar state (HHS) which require hospitalizations also increased among young adults during 2006 to 2015 [24]. A number of incriminating factors such as lower priority for self-care among younger adults, complexities of diabetes management, emotional changes during early adulthood, lower levels of HBA1c control due to inadequate follow-up care, higher rates of depression, and stress associated with major life transitions could be responsible for these findings [25,26,27].

We found that diabetes-associated hospitalizations significantly decreased among older population ≥60 years of age. The prevalence of diabetes increases with age and was as high as 29.5% among people aged 65 years and above during 2017–2018 [2]. In spite of increasing prevalence in diabetes, we observed a decreasing trend in diabetes-associated hospitalizations. Several factors could be responsible for these findings. A large majority of elderly adults are covered by some form of insurance including Medicare of Medicaid [28]. This could have improved their access and availability of primary care for better diabetes prevention and management, thus averting the need for hospitalizations. In addition, diabetes management strategies among older adults have increasingly become sophisticated with patient-centered guidelines, thereby improving care delivery and prognosis in this population [29].

Another finding in our study was increasing trends in diabetes-associated hospitalizations only among males, while no changes among females. In 2010, the hospitalization rates among males were lower and crossed the rates among females during 2013 and remained higher thereafter. However, the trends in the prevalence of diabetes increased for both males (14.5% to 15.8%) and females (10.2% to 13.2%) from 2009–2010 to 2017–2018, thereby making it difficult to explain the increasing trends in diabetes associated hospitalizations only among males [14]. The male and female US population also increased steadily at a constant rate during the study period. Since we used US population for calculating gender-specific hospitalization rates, the trend for males and females observed in our study could be solely attributed to the changes in the rates of hospitalization for males and females. However, it is unclear as to what could have influenced these trends and future studies should investigate it.

One of the major strengths of our study is that we used largest all payer inpatients data for this analysis. Since the NIS is representative of majority of the community hospitals in the US, our estimates have better accuracy and greater external validity. In addition, we used the most recently available national data on diabetes associated hospitalizations, thus showing the most recent trends. Irrespective of these strengths, our study has some limitations. Though administrative databases are meticulously quality assured, they are still susceptible to coding errors. ICD-9 and ICD-10 codes were used to identify diabetes associated hospitalizations and there could be some coding errors leading to misclassification bias. NIS database deleted all personal identifiers to ensure confidentiality procedures. Patients who were readmitted were considered as independent new admissions, thus obliterating the difference between index case and readmitted case. This could have led to overestimation of hospitalization rates. The NIS does not consider diabetes-associated hospitalizations in federal hospitals such as the Department of Defense, Department of Veterans Affairs, and Indian Health Service. Though these hospitals represent only a tiny fraction of the health care network, their non-inclusion could restrict the external validity of our study findings. In 2012, the NIS was redesigned for calculating more accurate national estimates. Although we used appropriate weights to account for this transition, it could have affected the results of this study. Similarly, the NIS contains ICD-9 codes until the third quarter of 2015, after which ICD-10 codes were used. Although we used necessary steps in the crosswalk between the ICD-9 and ICD-10 codes for identifying diabetes-associated hospitalizations, this could have affected some of our findings. Due to the drawbacks of crosswalk, we did not stratify diabetes into Type 1 and Type 2.

## 5. Conclusions

Using a nationally representative database, we found that diabetes-associated hospitalizations increased from 2010 to 2019. The most alarming finding is the increasing trend observed among younger males which could have long lasting implications for both patients and the healthcare system and become a major public health concern. Given that recent evidence suggests a reappearance of diabetes complications, the findings in our study portend a continuing increase in diabetes-associated hospitalizations in the future. The trends observed in our study emphasize the pressing need for interventions and policies for screening, preventing, and controlling diabetes at a younger age.

## Figures and Tables

**Table 1:** Comparison of demographic characteristics of diabetes hospitalizations between 2010 and 2019.

Variables	2010 (*n* = 7,241,873)	2019 (*n* = 8,373,830)	*p* Value
Age group, *n* (%)			<0.001
18–29 years	135,882 (1.9%)	151,790 (1.8%)	
30–39 years	290,512 (4.0%)	308,500 (3.7%)	
40–49 years	692,250 (9.6%)	652,305 (7.8%)	
50–59 years	1,334,476 (18.5%)	1,403,455 (16.8%)	
60–69 years	1,731,351 (24.0%)	2,136,320 (25.6%)	
70–79 years	1,659,355 (23.0%)	2,161,665 (25.9%)	
≥80 years	1,380,572 (19.1%)	1,541,200 (18.4%)	
Gender, *n* (%)			<0.001
Males	3,457,701 (47.8%)	4,286,635 (51.2%)	
Females	3,783,506 (52.2%)	4,086,755 (48.8%)	
Race, *n* (%)			0.433
White	4,108,081 (63.7%)	5,181,249 (63.2%)	
Black	1,247,733 (19.3%)	1,493,565 (18.2%)	
Hispanic	723,074 (11.2%)	990,055 (12.1%)	
Asian or Pacific Islander	152,989 (2.4%)	238,210 (2.9%)	
Native American	59,019 (0.9%)	70,190 (0.9%)	
Other	160,816 (2.5%)	228,430 (2.8%)	
Insurance, *n* (%)			<0.001
Medicare	4,390,287 (60.2%)	5,329,005 (63.3%)	
Medicaid	835,050 (11.5%)	1,094,935 (13.0%)	
Private	1,512,996 (20.8%)	1,486,175 (17.7%)	
Self-pay	308,403 (4.2%)	270,410 (3.2%)	
Other	241,733 (3.3%)	236,030 (2.8%)	
Income quartiles, *n* (%)			0.542
First quartile	2,332,361 (33.1%)	2,826,940 (34.3%)	
Second quartile	1,876,330 (26.6%)	2,154,509 (26.2%)	
Third quartile	1,617,042 (22.9%)	1,910,875 (23.2%)	
Fourth quartile	1,223,970 (17.4%)	1,340,491 (16.3%)	
Length of stay, median (IQR)	3.1 (1.6–5.8)	3.2 (1.6–6.0)	0.526
Diabetic retinopathy, *n* (%)	731,429 (10.1%)	820,635 (9.8%)	0.072
Diabetic nephropathy, *n* (%)	1,636,663 (22.6%)	1,758,504 (21.0%)	0.085
Diabetic neuropathy, *n* (%)	1,911,854 (26.4%)	2,152,074 (25.7%)	0.092
Lower-extremity amputations, *n* (%)	79,660 (1.1%)	83,738 (1.0%)	0.081

Note: Income quartiles are based on the median household income for patient’s ZIP Code. Abbreviation: IQR, interquartile range.

## Data Availability

Data used for this study are from the National Inpatient Sample, and this database is available publicly for purchase at: https://www.hcup-us.ahrq.gov/nisoverview.jsp (accessed on 12 June 2022).

## Supplementary Materials

The following supporting information can be downloaded at: https://www.mdpi.com/article/10.3390/jcm11226636/s1, Table S1. ICD-9-CM and ICD-10-CM codes for diabetes associated hospitalizations; Table S2. Trends in diabetes-associated hospitalizations stratified by age and gender. Table S3. Top ten primary diagnoses among hospitalizations with diabetes in 2010 and 2019.
