# Supplementary material for: Recent Trends in Diabetes-Associated Hospitalizations in the United States
Source: J Clin Med. 2022 Nov 9;11(22):6636. doi: 10.3390/jcm11226636 (PMC9698503; doi:10.3390/jcm11226636)
Supplement: Supplementary file 1 [file jcm-11-06636-s001.zip › jcm-2010793-supplementary.pdf]

Supplementary Table S1. ICD-9-CM and ICD-10-CM codes for diabetes associated hospitalizations

Variable	ICD-9-CM code	ICD-10-CM code
Diabetes	250.XX	E10.XXX, E11.XXX, and E13.XXX

Supplementary Table S3. Top ten primary diagnoses among hospitalizations with diabetes in 2010 and 2019

**Number**	**2010 (n=7,241,873)**		**2019 (n=8,373,830)**	
	**Reason**	**%**	**Reason**	**%**
1	Diabetes mellitus with complications	8.1	Diabetes mellitus with complications	10.2
2	Coronary artery disease	6.8	Congestive heart failure	9.6
3	Congestive heart failure	4.9	Coronary artery disease	7.2
4	Sepsis	3.2	Pneumonia	7.2
5	Acute kidney failure	3.1	Cellulitis and abscess of leg	6.1
6	Cellulitis and abscess of leg	2.8	Sepsis	6.0
7	Atrial fibrillation	2.8	Hypertensive heart disease	3.7
8	Pneumonia	2.1	Acute kidney failure	3.2
9	Osteoarthrosis	2.0	Urinary tract infection	2.9
10	Urinary tract infection	1.9	Cerebral artery occlusion	2.1

Supplementary Table S2. Trends in diabetes associated hospitalizations stratified by age and gender

Year	2010	2011	2012	2013	2014	2015	2016	2017	2018	2019	Relative change	P_{trend}
Overall	3079.0	3210.7	3125.9	3068.0	3059.6	3125.9	3156.5	3248.6	3269.2	3280.8	6.6%	0.028
Age group												
18-29 years	261.8	256.2	262.2	257.5	260.6	272.2	282.8	287.5	286.7	282.3	7.8%	<0.001
30-39 years	723.6	721.4	708.3	695.0	701.5	701.4	710.2	713.6	700.3	697.7	-3.6%	0.082
40-49 years	1590.0	1592.3	1573.4	1537.8	1545.3	1581.5	1610.3	1611.3	1615.2	1618.2	1.8%	0.098
50-59 years	3166.4	3237.9	3160.8	3111.1	3133.3	3174.4	3240.9	3294.7	3318.2	3314.6	4.7%	0.019
60-69 years	5867.1	6029.4	5816.5	5636.0	5548.2	5578.6	5599.6	5693.6	5652.0	5621.2	-4.2%	0.036
70-79 years	9962.7	10491.6	9979.3	9592.9	9327.2	9400.0	9317.3	9400.1	9302.0	9129.5	-8.4%	0.002
Gender												
Male	3028.6	3162.1	3101.2	3077.3	3087.8	3170.4	3238.9	3354.4	3408.8	3446.9	13.8%	<0.001
Female	3125.9	3256.0	3149.2	3058.5	3032.1	3082.5	3075.2	3148.0	3136.3	3122.6	-0.1%	0.529
Age group by gender												
18–29-year males	187.7	187.9	191.9	188.9	190.2	205.3	215.3	215.9	222.2	221.6	18.1%	<0.001
18–29-year females	337.6	326.5	335.2	328.7	333.9	342.0	353.2	362.4	353.9	345.6	2.4%	0.012
30–39-year males	629.7	634.1	620.2	611.1	610.4	608.5	630.2	640.7	633.3	638.9	1.5%	0.316
30–39-year females	817.1	808.0	796.2	778.5	792.4	794.5	790.2	787.0	768.1	757.2	-7.3%	0.001
40–49-year males	1614.4	1610.0	1600.3	1585.6	1583.7	1635.2	1671.8	1686.3	1702.9	1717.0	6.4%	0.002
40–49-year females	1565.1	1574.3	1546.8	1490.4	1507.0	1528.0	1548.4	1537.4	1528.6	1520.7	-2.8%	0.237
50–59-year males	3362.6	3450.1	3380.2	3359.1	3398.4	3451.0	3560.0	3642.3	3711.2	3728.8	10.9%	<0.001
50–59-year females	2979.5	3035.7	2951.8	2874.1	2879.7	2909.3	2932.2	2962.0	2941.3	2917.2	-2.1%	0.294
60–69-year males	6205.2	6356.7	6168.6	6040.1	5993.4	6068.5	6146.2	6297.3	6321.3	6339.8	2.2%	0.437
60–69-year females	5559.3	5731.4	5496.2	5268.1	5143.1	5131.9	5098.9	5147.5	5046.5	4971.2	-10.6%	<0.001
70–79-year males	10703.0	11279.2	10782.0	10449.0	10228.2	10340.1	10351.7	10475.9	10420.6	10288.0	-3.9%	0.035
70–79-year females	9361.0	9847.8	9320.1	8884.6	8578.8	8615.3	8446.5	8497.8	8361.2	8154.3	-12.9%	<0.001
≥80-year males	13740.8	14481.9	13865.2	13553.2	13302.1	13539.1	13387.6	13921.0	13937.1	13914.1	1.3%	0.742
≥80-year females	11340.5	11996.6	11311.9	10903.1	10595.9	10808.8	10467.9	10841.6	10754.0	10660.7	-6.0%	0.016
